# Active inference and psychodynamics: a novel integration with applications to depression and stress disorders

**DOI:** 10.3389/fpsyt.2025.1630858

**Published:** 2025-08-19

**Authors:** Hebert W. Harris

**Affiliations:** Private Practitioner, Arlington, VA, United States

**Keywords:** active inference, predictive processing, psychodynamic theory, second-order self, depression, PTSD, free energy principle, computational psychiatry

## Abstract

This paper introduces Active Intersubjective Inference (AISI), a novel framework that integrates psychodynamic theory with predictive processing to explain self-identity construction and psychopathology. AISI posits that the self emerges from recursive inferences about how others perceive us (second-order self), interacting bidirectionally with interoceptive processes. We map psychodynamic phenomena (e.g., transference, projection) onto neurocomputational mechanisms and apply AISI to Major Depressive Disorder (MDD) and Post-Traumatic Stress Disorder (PTSD), highlighting distorted second-order inference as a core dysfunction. Therapeutic implications include psychodynamic therapy, cognitive-behavioral approaches, and psychedelic-assisted treatments to enhance inference flexibility. AISI bridges psychodynamic insights with the NIMH Research Domain Criteria, offering a testable model for precision psychiatry and future clinical trials.

## 1 Introduction

Psychodynamic theories have long emphasized the centrality of relationships in shaping the self. However, they have lacked a neurobiological framework. Conversely, neuroscience and computational psychiatry have made substantial progress in modeling brain function as predictive inference. This paper aims to bridge these domains by introducing Active Intersubjective Inference (AISI), a framework that maps psychodynamic concepts onto a formal model of recursive prediction.

The AISI framework proposes that the self is constituted through active inferences about how others perceive us. We extract information about ourselves from the language and behavior of others, and we refer to the resulting predictive model as the second-order self. In contrast, the first-order model of self is derived directly from bodily sensations, including proprioception and physiological data. These direct, first-order models are termed interoceptive selves (1–4).

We view personal identity as arising from bidirectional interactions between the interoceptive and second-order selves. Interoceptive states influence our perceptions of social cues. At the same time, the threats, attitudes, and intentions of others, processed through AISI, shape the second-order self, which influences physiological responses (e.g., cortisol release) via top-down modulation of the interoceptive self. Conversely, bottom-up interoceptive signals (e.g., heart rate changes) update second-order self-models.

The prior interpersonal experiences that shape the second-order self are continuously updated through feedback from real and imagined interactions with others. Constructing second-order models from social, linguistic, and behavioral cues is complex and error-prone. It relies heavily on Bayesian priors derived from narrative memories that may date back to interpersonal interactions of early childhood.

AISI is a highly active inference process in which our language and behavior influence the inferences and behaviors of others in real time. We use the term intersubjective to emphasize that it involves the interactions of mental representations of self and other between two or more individuals.

The Free Energy Principle (FEP) holds that organisms behave in ways that minimize errors generated by active inference processes (5, 6). In this paper, we observe that applying the FEP to AISI may elucidate common psychodynamic processes such as projection and splitting while addressing many psychodynamic aspects of depression and stress-related disorders.

This paper focuses on MDD and PTSD to illustrate AISI’s applicability to trauma and depression. The paper is structured first to define the AISI model and its neuroanatomical underpinnings, then to apply it to clinically relevant psychodynamic processes, as well as to major depressive disorder (MDD) and post-traumatic stress disorder (PTSD). Finally, we consider therapeutic implications and outline a future research agenda grounded in computational psychiatry and AI-enhanced psychodynamic assessment.

## 2 Theoretical framework

### 2.1 Overview of active inference

The active inference framework, developed by Karl Friston and colleagues, extends predictive processing through the free energy minimization principle (5). Here, “free energy” is a mathematical concept that quantifies the difference between the brain’s internal model of the world and the actual state of the world as indicated by sensory data. In simpler terms, free energy is a measure of surprise or uncertainty.

According to this framework, biological systems are driven to minimize free energy through motor and sensory mechanisms. The brain constantly forms internal models that are updated to better match sensory input. Additionally, the brain initiates actions to alter sensory input in two ways: (1) changing perception, such as redirecting gaze to clarify ambiguous social cues (e.g., observing a facial expression), and (2) modifying the environment, such as engaging in behaviors to elicit specific social responses (e.g., smiling to evoke a positive reaction) (7, 8).

Precision-weighting is a key mechanism in active inference. Prediction errors are assigned weights based on their estimated reliability or “precision.” High-precision errors drive model updates, while low-precision errors may be effectively ignored. This precision-weighting mechanism allows the brain to flexibly balance reliance on prior models versus new information, depending on context and levels of uncertainty. For example, in a noisy social setting (high uncertainty, e.g., a crowded party with ambiguous facial expressions), the brain may assign low precision to sensory data, relying more on priors (e.g., expecting friendliness from known peers). Conversely, in a clear context (low uncertainty, e.g., a one-on-one conversation with distinct cues), sensory data receive higher precision, driving model updates (e.g., revising a prior of rejection when a smile is clearly observed). Uncertainty reflects the brain’s estimate of variability in sensory data or priors, distinct from but influencing the size of prediction errors, which are the mismatches between predictions and actual input (5). The relevance of information for reducing uncertainty is determined by its potential to minimize free energy, prioritizing reliable data (e.g., consistent social cues like repeated smiles) and contextually salient information (e.g., threat cues in dangerous settings or affiliative cues in bonding scenarios). Attention amplifies high-precision signals to optimize model updates (9).

The predictive hierarchy refers to layered brain processes where lower levels predict immediate sensory data (e.g., seeing a smile) and higher levels predict abstract states (e.g., others’ approval). For instance, in a social setting, low-level predictions about facial expressions feed into high-level predictions about social acceptance. Precision allocation across this hierarchy is understood as a form of attention that directs the system’s resources toward information considered most relevant for reducing uncertainty. Attention to one’s own predictions is possible, as individuals can reflect on their expectations (e.g., ‘I expect rejection’) and adjust them based on new evidence, a process central to second-order inference. Disruptions in precision allocation are implicated in various psychological conditions, ranging from hallucinations (overweighted prior beliefs) to heightened anxiety (overweighted threatening prediction errors) (10, 11).

To ensure the AISI framework is empirically testable, its predictions can be evaluated using computational models such as the Hierarchical Gaussian Filter (HGF; 12), which quantifies belief updating in social contexts, and neuroimaging techniques like fMRI to measure activity in regions supporting second-order inference (e.g., medial prefrontal cortex, anterior insula). Virtual reality (VR)-based tasks can further simulate social interactions to assess inference flexibility, as detailed in Section 6.1.

### 2.2 From individual to intersubjective prediction

In social contexts, the brain’s predictive architecture faces a unique challenge. We must model the mental states and beliefs of other agents who are actively forming predictions about us. This creates a reciprocal system in which each person’s predictions and actions provide sensory data for others’ predictions. Moreover, our actions are shaped not only by our models of others but also by our models of how others model us, creating nested, recursive prediction hierarchies that are characteristic of human intersubjectivity.

### 2.3 First-order and second-order inference

#### 2.3.1 First-order inference: modeling bodily states and observable behaviors

First-order inference operates on data derived from internal, physiological sources. It produces predictive models of autonomic, neuroendocrine, and proprioceptive states. At the highest levels, these models combine into a framework known as the interoceptive self (1–3). The interoceptive self integrates information about energy levels, appetite regulation, reward expectations, danger/anxiety signals, libido, and other biological drives that collectively shape mood states (13, 14). For example, in anxiety, first-order inference might amplify heart rate signals as predictions of imminent threat.

First-order inference is also employed to make predictive models of others’ directly observable behaviors, including speech, facial expressions, and actions. At this level, we model others as complex agents, but do not attempt to understand the intentionality underlying their behavior or comprehend their mental states. This process predominates during early development before Theory of Mind (ToM) emerges.

#### 2.3.2 Second-order inference: modeling others’ perceptions of the self

**Figure 1:**
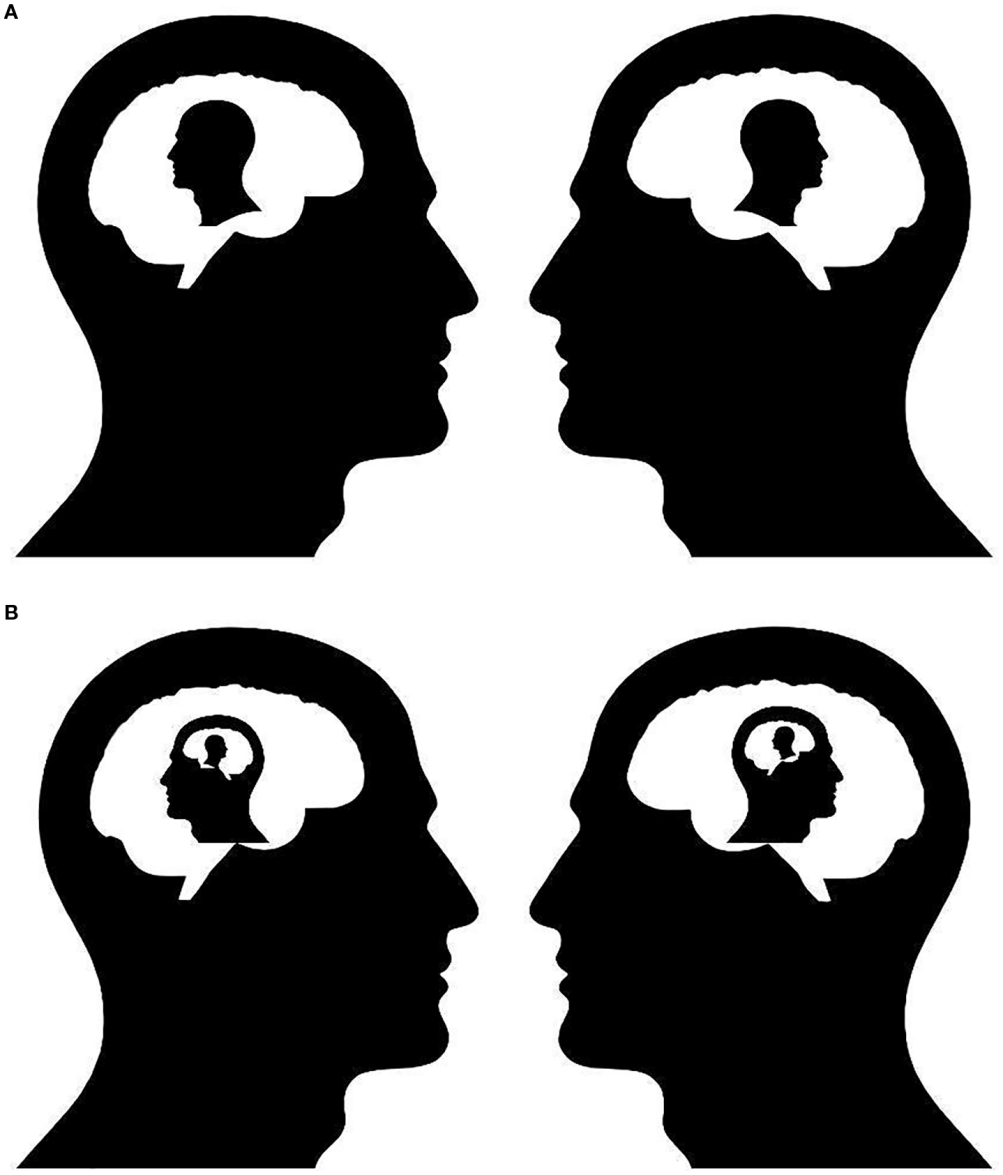
**(A)** First-Order Active Interpersonal Inference. This schematic illustrates an agent engaged in first-order active inference within a dyadic social interaction. The agent (left) uses sensory and contextual cues to generate predictions about the behavior, intentions, and emotional state of another person (right). However, the model remains egocentric; the agent does not represent how it is itself perceived by the other. In neurocomputational terms, this process recruits predictive coding hierarchies but does not yet involve higher-order recursive modeling. This form of inference supports basic empathy, behavioral synchronization, and social coordination but does not fully capture the reflexive dynamics of self-consciousness or interpersonal identity. **(B)** Second-Order Active Interpersonal Inference. This figure expands the model to depict second-order AISI, wherein the agent infers not only the other’s mental states but also how the other perceives the agent in return. This recursive loop—I model how you see me—enables the emergence of complex interpersonal emotions such as shame, pride, embarrassment, and trust. The diagram highlights the closed-loop architecture of second-order social inference, essential for self-conscious affect and reflective identity. Such modeling requires more complex generative architectures and may depend on the integration of medial prefrontal, temporoparietal, and default mode networks in the brain.

Once ToM development begins, second-order inference allows us to model others’ mental states, including how they perceive us—a process we refer to as recursive self-modeling. This generates predictive images of self and other (**Figures 1A, B**). In contrast to first-order inference’s focus on observable actions (e.g., Person A predicts Person B’s smile), second-order inference interprets underlying intentions (e.g., Person A infers that Person B’s smile indicates approval). Details of how Person B models Person A are disclosed through language and behavioral cues. From this information, Person A can construct inferential models of themselves. First-order and second-order inference continue in parallel after ToM develops, with first-order handling direct sensory and behavioral modeling while second-order adds interpretive layers for mental states.

The socially constructed self (second-order self) must align with other sources of information, such as interoception, which provides data on mood states and biological drives (1–3). The interoceptive components of self, such as physiological states and biological drives, interact bidirectionally with the second-order self to produce mood and affect, and to elicit physiological responses appropriate to social contexts. For instance, a perceived social threat (second-order) might trigger cortisol release (interoceptive), while physical fatigue (interoceptive) could bias perceptions of social rejection (second-order). This bidirectional loop operates as follows: top-down modulation from the second-order self (e.g., negative social feedback strengthens priors of rejection, increasing cortisol via the hypothalamus); bottom-up signals from the interoceptive self (e.g., physiological arousal signals danger, reinforcing negative second-order priors).

Self-experiences from activity, mastery, or situational performance (e.g., succeeding in a task) are integrated into the second-order self when interpreted through social feedback (e.g., praise from others) or internalized as self-evaluation, a process akin to Kohut’s mirroring (Section 3.5). These experiences contribute to priors of competence, distinct from but interacting with interoceptive signals.

**Figure 2:**
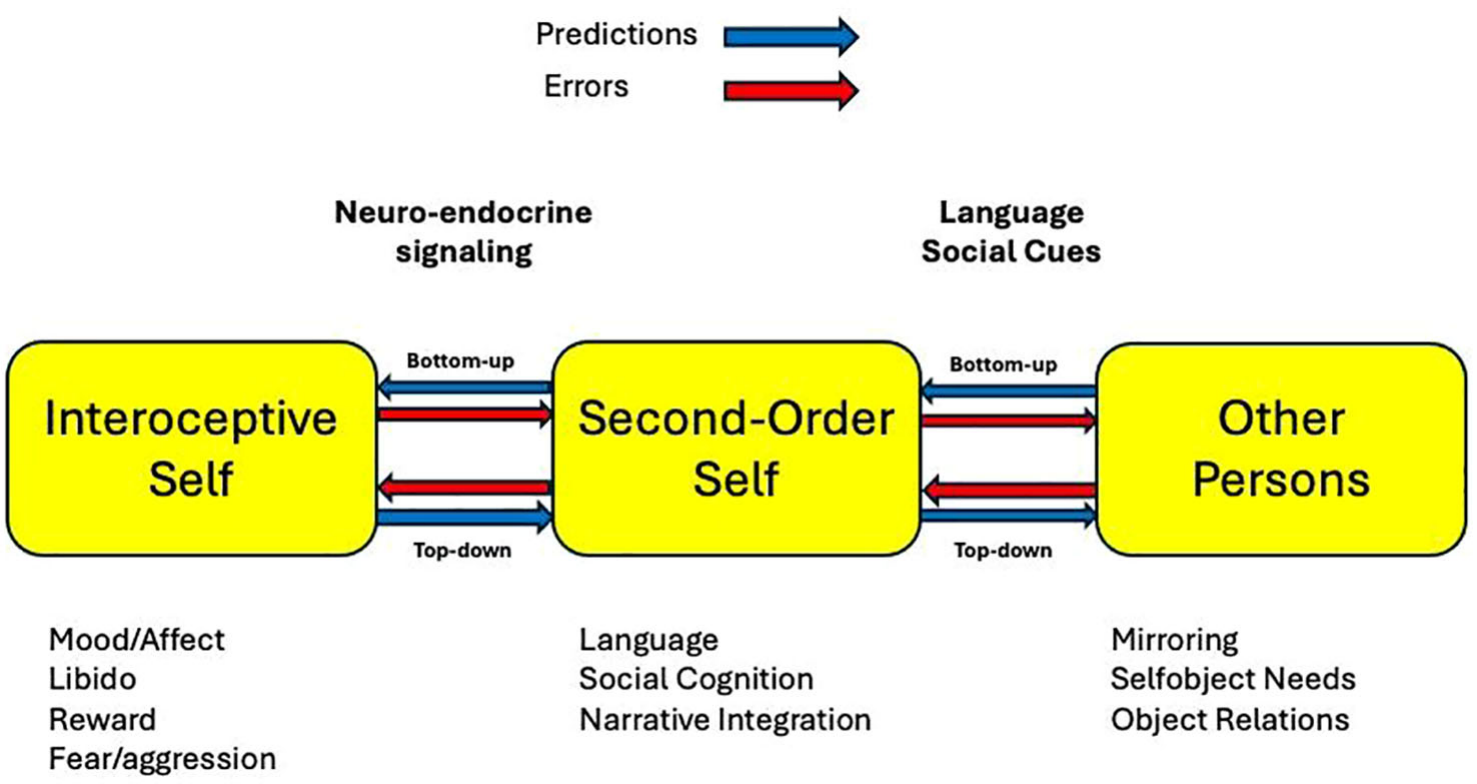
Interactions among interoceptive self, second-order self, and social others. This figure presents a schematic model illustrating the dynamic interplay among (1) the interoceptive self—which encodes bodily and affective signals such as hunger, fear, libido, and fatigue, (2) the second-order self, which encompasses reflective models of one’s identity and how one is perceived by others, and (3) external social agents, whose feedback and behavior recursively shape self-perception. Arrows depict bidirectional influences: neuroendocrine feedback (e.g., cortisol, oxytocin) modulates interoception, interpersonal feedback shapes second-order models, and recursive predictions about others’ perceptions regulate affective states. The diagram emphasizes how identity, affect regulation, and interpersonal behavior co-emerge through predictive coding across interoceptive and social levels of the self-model. To distinguish computational from anatomical elements (as per reviewer feedback), computational nodes (e.g., priors such as “others see me as worthless,” predictions like anticipating rejection, error signals from mismatches like unexpected praise) are implied in the flows; anatomical correlates (e.g., mPFC for second-order modeling, anterior insula for interoceptive bridging) are referenced in **Table 1**. Bottom-up flows represent sensory data updating models (e.g., physiological arousal influencing self-perception via error signals), while top-down flows involve priors shaping interpretation (e.g., expected rejection biasing neutral cues). For a detailed walkthrough with examples of first-order inference and trauma triggers, see Section 4. (Insert image3.png here).

As illustrated in **Figure 2**, these shared priors create a dynamic feedback loop between bodily experiences and social self-representations. Social interactions have profound effects on interoceptive processes. Experiences of social threat, rejection, or affiliative bonding trigger specific neuroendocrine cascades that alter physiological parameters, including heart rate variability, cortisol levels, and immune function (15, 16). These bidirectional interactions, central to AISI, explain how social disruptions cascade into physical symptoms in disorders like depression (see Section 4). Actions to alter sensory input are central to AISI, as they test predictions about others’ perceptions (e.g., seeking clarification in a conversation to resolve ambiguity in social cues).

### 2.4 The functional and evolutionary significance of higher-order inference

Cooperative activities—from hunting to childcare to cultural learning—depend on predicting others’ actions and understanding their expectations of us. The emergence of second-order inference capabilities likely created powerful selection pressures throughout human evolution. Individuals who could accurately model how group members perceived them would gain significant advantages in managing their social reputation, forming alliances, and navigating hierarchies. These capabilities may have co-evolved with language and ToM (see Section 2.6 for developmental details). In psychopathology, disruptions in this process contribute to relational instability (see Section 3).

### 2.5 Neuroanatomical substrate

The neuroanatomical basis of AISI involves interconnected brain regions that support bidirectional information flow between interoceptive and second-order processes, as shown in **Figure 2**. This figure illustrates the recursive structure of AISI, where self-representations are continuously shaped by social and physiological feedback. The interoceptive self integrates signals related to drives, reward, and homeostasis (e.g., motivation, libido, sleep, energy, appetite), which in turn modulate the second-order self through neuroendocrine pathways. A key assumption is that these regions enable prediction generation and feedback integration to update models.

Several large-scale brain networks overlap with this architecture, including the Default Mode Network (DMN), the Salience Network, and components of the Mirror Neuron System. Each region contributes to one or more functions necessary for intersubjective inference: modeling others’ mental states, integrating social feedback, mapping bodily states onto emotion, and maintaining narrative continuity of identity.

**Table 1** summarizes key regions implicated in AISI, focusing on those relevant to depression and trauma (detailed in Section 4). These include the medial prefrontal cortex (mPFC) for self-modeling, temporoparietal junction (TPJ) and posterior cingulate cortex (PCC) for social feedback, anterior cingulate cortex (ACC) for error monitoring, and anterior insula (AI) for interoceptive bridging. Notably, these regions are densely interconnected with subcortical structures (e.g., hypothalamus, amygdala), allowing the second-order self to modulate physiological states and vice versa. This substrate underpins the negotiation between internal drives and social expectations in AISI.

**Table 1:** Brain regions involved in AISI.

**Region**	**Known function**	**Rationale for involvement in AISI**
Medial Prefrontal Cortex (mPFC)	Self-referential processing; social cognition (17, 18)	Maintains hierarchical representations of self and others; supports second-order prediction models
Temporoparietal Junction (TPJ)	Social perspective-taking; predictive updating (19, 20)	Compares predicted and actual social feedback; essential for self-model revision
Posterior Cingulate Cortex (PCC)	Autobiographical memory; DMN integration (21, 22)	Integrates social feedback with narrative identity; maintains continuity of self
Anterior Cingulate Cortex (ACC)	Prediction error monitoring; conflict detection (23, 24)	Signals need for updating self-other models during interpersonal mismatch
Anterior Insula (AI)	Interoception; emotional salience (4, 25)	Links bodily states with emotional responses and social context
Precuneus	Mental imagery; social simulation (26, 27)	Simulates future social interactions and predicts others’ responses
Hippocampus	Episodic memory; predictive mapping (28, 29)	Encodes social interactions and supports learning from social prediction errors

### 2.6 Developmental trajectory of active intersubjective inference

Building on the distinction in Section 2.3, AISI develops alongside ToM. Through observation and interaction, toddlers (1–2 years) build predictive models of caregivers’ behaviors. However, these are primarily first-order models. Theory of Mind (ToM)—the ability to attribute mental states to others—develops through predictable stages (30). The milestone of understanding false beliefs (ages 4–5) enables children to build second-order models of themselves based on caregivers’ inferences. ToM and second-order inference likely co-develop, with ToM enabling the shift to recursive modeling; more research is needed to establish the precise time course.

Winnicott’s (31) concept of play offers a psychodynamic parallel to active inference. Play serves as a low-risk space for testing social hypotheses. Mastery experiences in play contribute to second-order priors by providing feedback on competence (e.g., succeeding in a game elicits caregiver praise, reinforcing self-models of capability). Winnicott’s (32) “good-enough mother” aligns with AISI by providing empathic feedback to calibrate inference during development.

Over time, the second-order self acquires greater stability and autonomy as it becomes anchored by narrative memories. Bayesian priors favor continuity in self-models. However, constructing an autonomous self requires caregiver support; failures may lead to pathology (Section 3.5).

### 2.7 Integration of personal identity

The information we use to construct second-order models of ourselves comes from others, who may present the developing child with divergent or even contradictory images that must be integrated into their self-model. The continuity and coherence of the child’s second-order self are relative to the frame of reference provided by their caretakers. The child’s sense of identity depends on the stable, consistent presence of others. This relativity makes the child vulnerable to changes in this frame of reference. While this may be an unavoidable part of development, when it becomes extreme, the child may experience distortions of self/other boundaries and displacement of identity. Distortions and displacements of the second-order self’s identity may manifest as projection, splitting, and other psychodynamic phenomena discussed in Section 3.

Over time, the second-order self acquires greater stability and autonomy as it becomes anchored by a growing body of narrative memories. The FEP and Bayesian inference drive us to predict that our next self will maintain continuity with our recent selves. However, the construction of a fully autonomous, enduring self may require considerable support from the child’s caregivers. If the child is allowed to test their autonomy while consistently receiving empathic, validating feedback, they can develop second-order models with increasing independence. Failures at this stage may lead to pathological development, as discussed in Section 3.5.

To some extent, the relativity of the second-order self persists throughout life. For example, the common experience of feeling like a different person in various social contexts or adopting collective identities of race, ethnicity, and nationality manifests the malleability of second-order identity (33). The nearly universal experiences of empathy and compassion may reflect displacements of identity with the transient, partial exchange of self with other. The integration of second-order models of the self into larger narratives is a process that continues throughout the entire lifecycle (34).

## 3 Psychodynamic phenomena as active intersubjective inference

AISI reframes psychodynamic processes as strategies for managing social uncertainty via free energy minimization. **Table 2** summarizes key phenomena, their AISI mechanisms, and examples, reducing overlap in the text below.

**Table 2:** Psychodynamic phenomena in AISI terms.

Phenomenon	AISI mechanism	Clinical example
Transference	Applying Bayesian priors from past relationships to new contexts; resistant updating due to high prior precision	Patient expects therapist criticism based on parental models, ignoring neutral cues
Projection	External attribution of ego-dystonic content to minimize self-model errors	Attributing anger to a colleague to avoid internal conflict
Splitting	Partitioning self-models into binary categories for computational efficiency	Viewing self/others as all-good or all-bad in borderline states
Projective Identification	Active induction of projected content in others via intersubjective loops	Inducing helplessness in therapist to confirm disowned vulnerability
Self Psychology (Narcissism)	Reliance on external mirroring due to inflexible second-order priors	Needing constant validation to sustain fragile self-coherence

### 3.1 Transference as Bayesian inference

Transference redirects past relational priors to current interactions (35). In AISI, these act as Bayesian priors for second-order models. Mismatches generate errors, but ingrained priors resist updates due to: (1) strong developmental weighting, (2) inflexibility, or (3) maladaptive error thresholds. For example, a patient might transfer abandonment fears onto the therapist, minimizing short-term uncertainty but perpetuating cycles. These inflexible priors are rigid beliefs that fail to account for alternative interpersonal scenarios (e.g., expecting hostility in all interactions, unable to consider positive alternatives). Therapeutic co-creation (36) minimizes dyadic free energy, sometimes reinforcing patterns.

### 3.2 Projection

Projection attributes unacceptable impulses externally (37). In AISI, it resolves ego-dystonic errors by displacing them to others’ models, reducing internal free energy. For example, a depressed individual projects self-loathing as others’ judgment, maintaining self-coherence at interpersonal cost.

### 3.3 Splitting

Splitting divides experiences into extremes (38). AISI views it as partitioning second-order self-models to avoid integration costs. Under stress, binary categories reduce uncertainty short-term but lead to oscillations (e.g., idealizing/devaluing in relationships). Neurobiologically, it involves poor valence network integration.

### 3.4 Projective identification

Projective identification induces others to enact projections (38). AISI breaks it into: disavowal, projection, active induction via intersubjective loops, and identification. This blurs self-other boundaries, as in trauma where victims induce rejection to confirm priors.

### 3.5 Self psychology in the AISI framework

Kohut’s (39) self psychology emphasizes self object needs in narcissism. In AISI, developmental failures lead to reliance on mirroring for second-order stability. Empathic therapy updates priors, enhancing flexibility. Neuroimaging shows altered insula/mPFC in subjects with narcissistic traits (40, 41).

## 4 Major depressive disorder and post-traumatic stress disorder through the lens of AISI

Both major depressive disorder (MDD) and post-traumatic stress disorder (PTSD) involve distortions in the modeling of the second-order self, the predictive representation of how others perceive one. As illustrated in **Figure 2** and supported by the neural structures listed in **Table 1**, the second-order self and interoceptive self are engaged in recursive, bidirectional inference loops. Dysfunction in either domain can cascade to the other, producing the range of affective, somatic, and cognitive symptoms observed in these conditions.

To clarify the model’s structure (as illustrated in **Figure 2**), let’s walk through its key steps. The interoceptive self generates bottom-up signals from physiological data (e.g., heart rate variability as error signals indicating arousal), which update priors in the second-order self (e.g., interpreting arousal as social threat). Conversely, top-down flows from the second-order self propagate predictions (e.g., “others reject me”) to shape interoceptive interpretation, minimizing overall prediction errors. First-order social inference operates in parallel, such as a smile eliciting a reciprocal smile via direct behavioral mirroring (e.g., mirror neuron activation) without engaging second-order mental state attribution. In trauma, a trigger like a sound can elicit both predictive processing (reactivating rigid second-order priors of vulnerability, leading to re-experiencing) and direct neuroendocrine changes (e.g., immediate amygdala-driven cortisol release, bypassing full inference). These bidirectional, recursive processes—grounded in free energy minimization—highlight how distortions cascade across levels in MDD and PTSD.

**Table 3** summarizes these interactions in normal versus pathological states, providing a quick reference for how first-order, second-order, and physiological responses align or diverge.

**Table 3:** AISI processes in social and traumatic contexts.

Process	Normal state	Pathological state (e.g., MDD/PTSD)
First-Order Inference (Observable Behaviors/Physiological Signals)	Direct mirroring of behaviors (e.g., smile elicits reciprocal smile via mirror neurons); flexible updating of physiological signals (e.g., heart rate as temporary arousal).	Rigid behavioral responses (e.g., hypervigilance to neutral cues); overamplification of signals (e.g., persistent arousal misinterpreted as threat).
Second-Order Inference (Mental State Modeling)	Recursive updating of self-models based on social feedback (e.g., praise adjusts priors of self-worth); balanced precision weighting.	Distorted priors resist update (e.g., rigid negative self-view despite positive feedback); over precision on threat/rejection.
Physiological Responses (Neuroendocrine/Interoceptive)	Bidirectional adaptation (e.g., social bonding reduces cortisol); error minimization maintains homeostasis.	Cascading dysregulation (e.g., trauma trigger causes direct amygdala-cortisol surge + predictive re-experiencing); chronic hyperarousal or shutdown.
Interaction Example	Neutral sound processed as benign; social cue integrates bottom-up data with top-down priors for adaptive response.	Sound triggers dual paths: direct neuroendocrine spike (e.g., fear response) and predictive reactivation (e.g., flashback), amplifying errors.

### 4.1 MDD and the self

From a predictive coding perspective, MDD involves maladaptive models of the interoceptive self, including dysfunctions in systems governing sleep, appetite, energy, libido, and reward-seeking (42). We extend this account to incorporate distorted second-order self-models, informed by psychodynamic theory.

Classic psychodynamic models of depression emphasize the role of internalized object relationships. Freud’s “Mourning and Melancholia” (43) described depression as a response to unconscious loss and identification with a punitive object. Klein, Bowlby, and Jacobson further elaborated on how object loss and ambivalent attachment contribute to depressive states. Blatt (44) distinguished between introjective depression, focused on self-criticism and guilt, and anaclitic depression, centered on feelings of abandonment and helplessness.

The AISI framework situates these perspectives within a computational model of second-order inference. Vulnerability to depression emerges from:

1. The loss of attachment figures or self objects who contributed positive second-order representations leads to a deficit in affirming self-models, producing symptoms associated with anaclitic depression (44, 45). These include feelings of abandonment, helplessness, and interpersonal dependency.
2. Internalization of critical or punitive object representations generates persistently negative second-order priors, manifesting as introjective depression with themes of guilt, inadequacy, and self-criticism.

These distorted second-order priors exert top-down suppressive effects on interoceptive prediction, leading to diminished energy, motivation, appetite, and reward-seeking. This state may serve an adaptive function—minimizing energy expenditure in anticipation of social futility, akin to a metabolic “hibernation” (46).

Mood emerges as a complex construct reflecting predictive modeling across multiple domains. Depressive mood states appear to represent the integration of predictions about energy availability, sleep-wake cycles, appetite regulation, libido, reward anticipation, anhedonia, and crucially, predictions of social rejection and social defeat (47–49). The social defeat stress model in rodents provides a compelling parallel to AISI dysfunction, where repeated social subordination leads to lasting alterations in both social behavior and physiological markers of depression (50).

Disruptions in interoceptive inference—due to chronic illness, inflammation, or dysregulated neurochemistry—may influence second-order models of the self. Low precision in interoceptive prediction increases the likelihood of misattribution (e.g., interpreting fatigue or somatic discomfort as signs of inadequacy or failure), reinforcing maladaptive second-order predictions (51; Gilbert et al., 2022). These bidirectional prediction processes, outlined in Section 2.3, demonstrate how disruptions in one domain cascade to affect the other, creating self-perpetuating cycles of dysfunction between the interoceptive self, the second-order self, and relations with others.

The AISI framework provides a unifying account for various depressive presentations. Melancholic depression, characterized by severe anhedonia and psychomotor changes, can be understood as pathologically rigid negative second-order priors combined with substantially disrupted interoceptive prediction. Atypical depression, with its altered mood reactivity and rejection sensitivity, reflects hypervigilant second-order inference systems that overweight potential social threats. These diverse presentations emerge from different patterns of dysfunction in the recursive loops between interoceptive and second-order inference systems.

Functional neuroimaging studies of MDD support this bidirectional model (52). Altered activity and connectivity have been consistently observed in regions central to AISI, including the mPFC, anterior insula, and ACC (52–54). In depression, positive social feedback often fails to update negative second-order self-perceptions, as they generate insufficient prediction error to override established negative priors.

In severe depression, suicide may represent a terminal outcome of unbearably negative second-order inference. When an individual’s negative self-models become so rigid that no corrective experiences can update them, the psychological pain may become intolerable.

### 4.2 Trauma and the second-order self

Post-traumatic stress disorder (PTSD) is characterized by exposure to actual or threatened death, serious injury, or sexual violence, followed by distinct symptom clusters: intrusive memories, avoidance behaviors, negative alterations in cognition/mood, and heightened arousal/reactivity (55). Complex PTSD (C-PTSD) includes additional disturbances in self-organization, emotion regulation, and interpersonal functioning (56).

Psychodynamic theories have long emphasized how trauma alters self-experience, noting the internalization of the perpetrator’s degrading view of the victim, the collapse of representational boundaries, and the inability to integrate traumatic experiences into a coherent self-narrative (57–59).

#### 4.2.1 PTSD and predictive models of the self

The interoceptive self plays a central role in PTSD symptomatology. Hyperarousal, sleep disturbances, and exaggerated startle responses reflect dysregulated predictions at the interoceptive level. These manifest physiologically through heightened sympathetic activity, elevated baseline cortisol, and disrupted hypothalamic-pituitary-adrenal axis functioning (60). Fear-potentiated startle—the exaggerated acoustic startle response in threatening contexts—provides a particularly well-documented example of altered predictive processing, with robust evidence from both human and animal studies (61, 62).

Within the AISI framework, trauma also produces enduring distortions in second-order self-models. In many cases, trauma involves a perpetrator who imposes a hostile and degrading image of the victim. This model, transmitted through coercion, fear, or shame, may override the victim’s preexisting second-order self-model, particularly if the trauma occurs during critical developmental stages. Once internalized, this hostile second-order model becomes a rigid prior, generating persistent predictions of vulnerability, worthlessness, and social rejection.

Triggering events in the present may reactivate these priors, leading to an intrusion of traumatic memories and associated affective states. Avoidance behaviors can be understood as active inference strategies to minimize exposure to stimuli that would generate high prediction errors against these trauma-informed models. The chronic hypervigilance in PTSD reflects an over precision of threat-related priors in second-order inference, where even neutral social cues are interpreted as confirmatory evidence of danger or rejection.

The bidirectional link between second-order and interoceptive selves explains PTSD’s somatic symptoms. A second-order model of the self as ‘damaged/dangerous/worthless’ maintains chronic stress system activation, perpetuating the physiological dysregulation characteristic of PTSD.

Neuroimaging studies support this model. PTSD has been associated with dysregulation in the medial prefrontal cortex (mPFC), anterior cingulate cortex (ACC), hippocampus, and anterior insula—regions implicated in both interoceptive inference and self-referential processing (63, 64). Reduced mPFC-amygdala connectivity may impair updating of trauma-related second-order priors, perpetuating hypervigilance and maladaptive threat perception in social contexts (24, 65).

#### 4.2.2 Complex PTSD and identification with the aggressor

Complex PTSD (C-PTSD) stems from prolonged, repeated trauma exposure, particularly during developmental periods (56, 66). Its distinctive features include profound disturbances in self-organization manifesting as persistent negative self-concept, affective dysregulation, and enduring difficulties in relationships. These identity disturbances often involve feelings of emptiness, chronic shame, and fundamental alienation from one’s authentic self (67, 68). Therapeutic approaches like prolonged exposure, while effective for uncomplicated PTSD and some C-PTSD cases (69), may require adaptation for C-PTSD due to its distinct disturbances in self-organization and interpersonal functioning.

The disturbances in identity found in C-PTSD can be understood through AISI as the internalization of the aggressor’s degrading second-order model of the victim, a process Ferenczi (57) termed “identification with the aggressor.” In prolonged trauma, the victim’s second-order self may incorporate the perpetrator’s hostile perceptions to minimize free energy in an unpredictable environment. This adaptive strategy during trauma becomes maladaptive post-trauma, leading to fragmented self-models and relational instability.

## 5 Integration with research domain criteria

The NIMH Research Domain Criteria (RDoC) initiative aims to reconceptualize mental disorders in terms of brain-behavior dimensions (70). While RDoC has advanced biological psychiatry, it has struggled to incorporate the subjective experience and meaning-making processes central to psychodynamic theories. We propose that the AISI framework can bridge these perspectives, demonstrating how traditional psychodynamic concepts can be reformulated in terms of modern computational neuroscience.

### 5.1 RDoC and AISI: complementary frameworks

RDoC organizes psychopathology along functional domains (negative valence, positive valence, cognitive systems, social processes, arousal/regulatory systems) examined across multiple levels from genes to behavior. The AISI framework’s emphasis on bidirectional interactions between interoceptive processes and social inference maps naturally onto these domains:

- Negative Valence Systems: AISI reinterprets sustained threat and loss constructs as maladaptive second-order inference patterns, where overly precise prior beliefs about rejection or worthlessness drive affective disturbances.
- Positive Valence Systems: Reward processing disruptions are viewed as predictive consequences of negative second-order models, where anticipation of social rejection diminishes reward-seeking behavior.
- Social Processes: Social communication and perception difficulties reflect distortions in recursive second-order modeling.
- Arousal/Regulatory Systems: Emotion regulation impairments may reflect maladaptive precision weighting in predictive hierarchies, a computational reframing of defensive mechanisms.

Recent work by Banaraki et al. (71) has similarly proposed integrating predictive processing with RDoC domains, focusing on cognitive systems. Our approach extends this integration to encompass psychodynamic insights about self-organization and interpersonal functioning.

### 5.2 Depression through an integrated lens

As detailed in Section 4.1, depression involves distorted modeling of both interoceptive states and social perceptions. The AISI-RDoC integration offers new insights into traditional psychodynamic depression subtypes, where positive- and negative valence systems are involved:

Anaclitic depression—characterized psychodynamically by dependency and abandonment fears (44)—can be reframed as hypersensitive second-order inference about social acceptance coupled with low precision of interoceptive prediction. This computational profile explains both the intense need for external validation and difficulty regulating emotions independently.

Introjective depression—marked by self-criticism and perfectionism in psychodynamic theory—represents rigid high-precision second-order models of social judgment that resist updating despite contradictory evidence. This corresponds to RDoC’s sustained loss construct, where loss-related neural circuits maintain persistent activation patterns (81).

Neurovegetative symptoms reflect the interoceptive consequences of these maladaptive second-order models. Anhedonia emerges not simply from reward circuit dysfunction (RDoC’s positive valence systems) but from active inference processes where:

1. Second-order predictions of social rejection increase the expected free energy of reward-seeking behaviors.
2. This leads to adaptive withdrawal to minimize prediction errors.
3. Consequently, dopaminergic signaling, which encodes the precision of reward prediction, is downregulated.

This integrative view explains why pharmacological approaches targeting monoaminergic systems may restore hedonic capacity without addressing the underlying social prediction models driving depression (72).

### 5.3 PTSD through an integrated lens

Building on Section 4.2, the AISI framework reinterprets PTSD across multiple RDoC domains:

In the negative valence domain, sustained threat activation represents rigid trauma-informed priors that chronically bias perception toward danger detection. The “overgeneralization of conditioned fear” described in RDoC can be reinterpreted through AISI as overgeneralization of trauma-related second-order inference to non-threatening interpersonal contexts.

The distinction between acute stress disorder and PTSD reflects different stages of inferential adaptation:

1. Acute responses feature low precision of prior beliefs and high precision of sensory/interoceptive errors—an adaptive state that facilitates rapid model updating after unexpected danger.
2. Chronic PTSD develops when trauma-related models become entrenched as high-precision priors that resist revision, biasing perception toward threat detection across contexts.

## 6 Future directions

The AISI framework opens up numerous opportunities for exploratory research and clinical innovation. Here, we elect to emphasize a significant unmet need: the development of technologies that can facilitate efficacy studies of psychodynamic treatments.

Despite a long tradition of clinical observation and case-based research, psychodynamic interventions remain underrepresented in large-scale clinical efficacy trials. Several challenges hinder progress: the high degree of personalization in treatment, the lack of consensus on therapeutic mechanisms, and the limited availability of sensitive outcome measures.

AISI may provide a novel foundation for overcoming these barriers. By positing recursive inference, particularly second-order cognitive flexibility, as a transdiagnostic mechanism underlying various forms of psychopathology, the framework identifies a common therapeutic target that can be implemented across diagnoses and therapeutic modalities.

Several specific, testable hypotheses emerge from the AISI framework that could guide future research:

1. Individuals with depression will show reduced second-order inference flexibility, measurable through computational tasks that assess belief updating about how others perceive them, with greater rigidity correlating with depression severity.
2. PTSD symptoms will correlate with specific patterns of second-order prediction errors, particularly overestimation of negative social evaluation and underestimation of social support availability.
3. Successful psychotherapy should demonstrate quantifiable increases in second-order inference flexibility, detectable through pre- and post-treatment assessments using the Hierarchical Gaussian Filter or similar computational tool.
4. Precision-weighting abnormalities in second-order inference can be pharmacologically modulated, with serotonergic and dopaminergic agents showing differential effects on different aspects of social prediction updating.
5. Virtual reality-based social interaction paradigms will reveal distinct second-order inference signatures for different personality disorders, providing objective biomarkers for treatment response.

Emerging technologies in computational psychiatry and AI provide promising tools for testing and refining this model. One such tool is the Hierarchical Gaussian Filter (HGF), a generative model that simulates belief updating under uncertainty (12). HGF can be applied to behavioral data from social inference tasks to estimate parameters like learning rates and precision weighting, offering quantitative measures of second-order flexibility.

We also propose that therapy can be understood as a coupled inference system in which the therapist and patient recursively update their models of each other. These models can be simulated to test how various therapeutic stances (e.g., empathic mirroring *vs*. interpretive confrontation) shape model convergence and belief revision.

Recent advances in AI-based psychodynamic assessment offer complementary approaches. Hoermann et al. (73) demonstrated that natural language processing algorithms can identify patterns of interpersonal reasoning and second-order perspective-taking in clinical interviews. Similarly, Liébana et al. (74) used interactive AI systems to elicit and quantify social inference flexibility in real-time simulated dialogues. Their findings showed a strong correlation between AI-generated metrics and clinician ratings of interpersonal functioning. Future research must address ethical challenges, such as ensuring equitable access to AI-driven assessments and minimizing risks in psychedelic trials, as discussed in Section 5. Additionally, while AISI emphasizes social and interoceptive contributions to self-experience, it could be extended to incorporate non-social sources like mastery or performance in future work.

These developments suggest that the AISI framework may help integrate diverse research programs—ranging from classical psychoanalysis to contemporary computational modeling—into a unified empirical project. By identifying second-order cognitive flexibility as a mechanistic target and linking it to quantifiable parameters, AISI provides the groundwork for precision psychodynamic psychiatry.

### 6.1 Empirical approaches to testing AISI

The AISI framework generates testable hypotheses that can be evaluated using computational, behavioral, and neuroimaging methods. Below, we outline specific experimental paradigms to assess AISI’s predictions about second-order inference flexibility and its role in psychopathology:

1. Neuroimaging Studies: Functional MRI (fMRI) can be used to measure activity and connectivity in AISI-related brain regions (e.g., medial prefrontal cortex [mPFC], anterior insula, temporoparietal junction [TPJ]) during social feedback tasks. For example, participants could engage in a task where they receive positive or negative evaluations from virtual agents, allowing researchers to examine how prediction errors in second-order inference correlate with activity in these regions. Reduced mPFC-anterior insula connectivity in MDD or PTSD patients would support AISI’s hypothesis of disrupted social inference (63).
2. Computational Modeling with Hierarchical Gaussian Filter (HGF) 80: The HGF (12) can quantify belief updating in second-order inference. In a behavioral task, participants could predict others’ perceptions of them based on ambiguous social cues (e.g., neutral facial expressions). HGF parameters, such as learning rate and precision weighting, could reveal reduced inference flexibility in MDD (e.g., rigid negative priors) or PTSD (e.g., overestimation of threat). Comparing these parameters across clinical and control groups would validate AISI’s mechanistic claims.
3. Virtual Reality (VR)-Based Paradigms: VR environments can simulate dynamic social interactions to test AISI’s predictions about recursive self-modeling. For instance, a VR task could present participants with avatars delivering ambiguous feedback, measuring how quickly participants update their second-order self-models in response to new information. Such paradigms, inspired by Liébana et al. (74), could quantify inference flexibility and identify biomarkers for treatment response in depression or trauma-related disorders.

These paradigms, summarized in **Table 4**, offer feasible approaches to test AISI’s hypotheses. While large-scale neuroimaging studies may be resource-intensive, pilot studies using HGF or VR tasks provide accessible starting points for empirical validation.

**Table 4:** Proposed experimental paradigms for AISI.

Method	Hypothesis	Task description	Expected outcome	Relevant disorders
fMRI Neuroimaging	AISI-related regions (mPFC, anterior insula, TPJ) show altered activity during second-order inference in MDD/PTSD.	Participants receive positive/negative social feedback in a task, with fMRI measuring regional activity and connectivity.	Reduced mPFC-anterior insula connectivity in MDD/PTSD, reflecting impaired inference flexibility.	MDD, PTSD
Hierarchical Gaussian Filter (HGF)	Reduced second-order inference flexibility correlates with symptom severity.	Behavioral task where participants predict others’ perceptions based on ambiguous cues, modeled with HGF.	Lower learning rates and higher prior precision in MDD/PTSD patients *vs*. controls, indicating rigid self-models.	MDD, PTSD, Personality Disorders
VR-Based Social Interaction	Inference flexibility predicts treatment response in social inference tasks.	VR task with avatars delivering ambiguous feedback, measuring belief updating speed.	Faster belief updating in healthy controls *vs*. slower updates in MDD/PTSD, with improvements post-therapy.	MDD, PTSD

### 6.2 Treatment implications

While the therapeutic applications of AISI are promising, they remain speculative and will require further empirical research to validate their clinical utility. The AISI framework identifies three independent axes along which patients can be classified: second-order inference flexibility, interoceptive-social coupling strength, and prior precision weighting. These axes, when integrated with RDoC constructs, offer a mechanistic basis for diagnosis, severity assessment, and treatment selection tailored to underlying psychopathological processes.

For instance, psychodynamically oriented treatments—such as empathic attunement, object relational approaches, transference analysis, or interpersonal therapy—may be particularly indicated for deficits in second-order inference flexibility or maladaptive prior precision weighting. These interventions provide corrective interpersonal experiences that generate prediction errors, facilitating the updating of rigid self-models.

In contrast, modalities like cognitive-behavioral therapy (CBT), exposure therapy, or dialectical behavior therapy (DBT) could be selected for disruptions in interoceptive-social coupling strength, as they target the integration of bodily signals with social cognition through structured behavioral experiments and emotion regulation techniques.

Pharmacotherapies present additional opportunities. Psychedelics, such as psilocybin and MDMA, show potential for addressing second-order inference flexibility and prior precision weighting by temporarily reducing the rigidity of high-level priors (75–77). Other pharmacotherapies, including serotonergic and dopaminergic agents, should be investigated for their differential effects on these three axes, potentially modulating precision weighting in predictive hierarchies (78, 79).

For outcome prediction, AISI parameters—such as baseline second-order inference rigidity—could forecast treatment response. These might be measured through computational tasks (e.g., HGF-based assessments) or AI-driven tools that quantify interpersonal reasoning in clinical interviews (73). This approach supports precision psychiatry by enabling mechanism-based interventions, though prospective studies are needed to confirm these predictions.

## 7 Conclusion

The Active Intersubjective Inference framework provides a novel and integrative understanding of mental function and dysfunction. By suggesting that the brain constructs not only first-order models of bodily and environmental states but also second-order models regarding how one is perceived by others, it links phenomenological experiences of self, affect, and identity with the neurocomputational principles of predictive coding.

This framework sheds light on the pathogenesis of conditions such as MDD and PTSD, where recursive self-models and interoceptive processes become rigid, maladaptive, or fragmented. It also explains the relational instability observed in personality disorders, where second-order predictions lack coherence or resilience.

Crucially, AISI suggests that treatment may enhance second-order cognitive flexibility—whether through empathic attunement in psychodynamic therapy, corrective experiences in CBT, or pharmacological modulation of precision weighting. Psychedelics and AI-driven assessments serve as powerful adjuncts for promoting plasticity and personalizing interventions.

By grounding psychodynamic insights in the formal logic of inference, AISI creates a common language for dialogue between clinicians, neuroscientists, and computational modelers. AISI advances psychodynamic understandings of trauma and depression by providing testable computational mechanisms, bridging neurobiological and psychological perspectives in ways that can inform both therapeutic practice and empirical research. This may finally allow for a truly integrative psychiatry—one that respects the depth of subjective experience while embracing the rigor of mechanistic explanation.

## Data Availability

The original contributions presented in the study are included in the article/supplementary material. Further inquiries can be directed to the corresponding author.
